# Data Quality Degradation on Prediction Models Generated From Continuous Activity and Heart Rate Monitoring: Exploratory Analysis Using Simulation

**DOI:** 10.2196/40524

**Published:** 2023-05-03

**Authors:** Jason Hearn, Jef Van den Eynde, Bhargava Chinni, Ari Cedars, Danielle Gottlieb Sen, Shelby Kutty, Cedric Manlhiot

**Affiliations:** 1 Blalock-Taussig-Thomas Heart Center Johns Hopkins University Baltimore, MD United States

**Keywords:** wearables, time series, data reliability, prediction models, hear rate, monitoring, data, reliability, clinical, sleep, data set, cardiac, physiological, accuracy, consumer, wearables, device

## Abstract

**Background:**

Limited data accuracy is often cited as a reason for caution in the integration of physiological data obtained from consumer-oriented wearable devices in care management pathways. The effect of decreasing accuracy on predictive models generated from these data has not been previously investigated.

**Objective:**

The aim of this study is to simulate the effect of data degradation on the reliability of prediction models generated from those data and thus determine the extent to which lower device accuracy might or might not limit their use in clinical settings.

**Methods:**

Using the Multilevel Monitoring of Activity and Sleep in Healthy People data set, which includes continuous free-living step count and heart rate data from 21 healthy volunteers, we trained a random forest model to predict cardiac competence. Model performance in 75 perturbed data sets with increasing missingness, noisiness, bias, and a combination of all 3 perturbations was compared to model performance for the unperturbed data set.

**Results:**

The unperturbed data set achieved a mean root mean square error (RMSE) of 0.079 (SD 0.001) in predicting cardiac competence index. For all types of perturbations, RMSE remained stable up to 20%-30% perturbation. Above this level, RMSE started increasing and reached the point at which the model was no longer predictive at 80% for noise, 50% for missingness, and 35% for the combination of all perturbations. Introducing systematic bias in the underlying data had no effect on RMSE.

**Conclusions:**

In this proof-of-concept study, the performance of predictive models for cardiac competence generated from continuously acquired physiological data was relatively stable with declining quality of the source data. As such, lower accuracy of consumer-oriented wearable devices might not be an absolute contraindication for their use in clinical prediction models.

## Introduction

There are numerous devices available within hospital settings to continuously monitor patients’ physiological signs, track disease progression, or perform diagnostics. In recent years, there has been increased interest in extending the use of these devices to the outpatient setting [1]. For this purpose, such devices are well accepted by patients and physicians and are generally seen as an important component of future clinical protocols [1-3]. There are two major categories of outpatient monitoring device: medical-grade and consumer-oriented devices. The advantages of consumer-oriented devices are obvious—they are often less expensive, they are ubiquitous, and their use is not dependent on a person’s medical need. Moreover, such devices allow data to be acquired continuously, passively, and without the need for standardized protocols or deviation from normal daily routine—all factors that have been associated with reduced adherence to outpatient monitoring [4]. However, contrary to their medical-grade counterparts, which are made to replicate the functionality and reliability of in-hospital devices, consumer-oriented devices are built for other purposes, which may affect the quality of the data that they generate. Numerous studies have shown limited equivalence between the data generated from consumer-oriented devices and the data acquired using standardized protocols involving their medical-grade counterparts [5-8]. Given these limitations, and although the reliability of data from newer consumer-oriented devices has greatly increased, some have advocated caution in the use of consumer-grade wearable devices for clinical monitoring when the intent is to integrate the data directly into care management [9]. One emerging application of data generated from physiological-monitoring devices is using them to produce features in prediction models based on machine learning algorithms, as opposed to the detection of abnormalities and direct integration in care management pathways [10]. In this context, the effect of decreasing data quality and reliability has not been previously studied. In this study, our aim was to investigate the practicality of using consumer-grade monitoring devices in medical care by determining the effect of various common forms of time-series data degradation on the performance of the predictive models generated using those data.

## Methods

### Study Data

Data were obtained from the open-access Multilevel Monitoring of Activity and Sleep in Healthy People (MMASH) data set made available by Rossi et al [11] on PhysioNet [11,12]. These data were collected through a collaboration between BioBeats and researchers at the University of Pisa. The MMASH data set includes 1 day of activity and sleep data for 22 healthy young adult males. During the 24-hour data collection period, the participants wore a heart rate (HR) monitor (Polar H7) and an activity monitor (ActiGraph wGT3X-BT). The participants were also asked to record specific times and categories (eg, sleeping, sitting, and heavy exercise) of activity that they performed throughout the day [11]. For the purposes of this secondary analysis, pertinent raw data from this data set included demographic information, step count data from the activity monitor, beat-to-beat intervals (or N-N intervals [NNIs]) from the HR monitor, and activity categories as reported by participants. One of the participants was removed from the analysis due to incomplete information.

### Data Processing and Feature Extraction

All MMASH data were downloaded from PhysioNet. The first 5 minutes of the activity monitor and HR monitor data were removed to account for the initial placement and adjustment of the devices. Per-minute step count (PMSC) was calculated for each participant by summing the number of steps taken in sequential 60-second periods. PMSC data during sleep hours for all of the participants was removed from the analysis, as these time series were flat and did not provide predictive value. Three features were used to summarize the PMSC data for each participant, which were maximum PMSC, median PMSC, as well as 25th and 75th percentiles in PMSC. HR measurements were calculated as the quotient of 60 divided by the NNI values. These HR values were then transformed to beats per minute by averaging them in sequential 60-second periods. Any values in the HR time series less than 35 beats per minute were set equal to 35 (lowest plausible value in the data set). Next, various HR variability statistics were calculated for each participant based on the Python code published by the research team responsible for the MMASH data set [13]. Time-domain features were calculated relating to NNIs (median, mean, standard deviation, root mean square, range, and percentage of differences greater than 50) and HRs (maximum, minimum, mean, and standard deviation) recorded by the HR monitor. Various frequency-domain features relating to HR variability were also computed for each participant, such as very low-frequency power (0.003 to 0.04 Hz), low-frequency power (0.04 to 0.15 Hz), high-frequency power (0.15 to 0.40 Hz), ratio of low-frequency power to high-frequency power, normalized low-frequency power, and normalized high-frequency power. Lastly, several features were extracted from a Poincaré plot of the NNIs, as follows: standard deviation of a projection onto the line perpendicular to the line of identity, standard deviation of the projection onto the line of identity, and the ratio of these two standard deviations [13].

Two R packages, *tsfeatures* and *tsfeaturex*, were employed to extract various higher-dimensional features from the time-series data including per-minute HR, NNIs, and PMSC. The extracted features included autocorrelation, partial and differential autocorrelations, probabilities of acute changes when the time series is lagged, and time series quantiles. A total of 87 features were extracted for each time series feature using these packages.

### Study Outcome

For each patient, we calculated the cardiac competence index (CCI) to be used as the target for prediction models. The CCI used in this study is based on the concept for cardiac competence limit proposed by Wu et al [14]; however, it was modified to be calculable with the data available in this study. In short, using activity information, data were isolated for periods of rest (ie, lying down or sitting) and periods of heavy exercise. Baseline HR was defined as the minimum of a rolling 2-minute mean of HR during periods of rest, whereas peak HR was calculated as the maximum of a rolling 2-minute mean of HR during heavy exercise (ie, the modification from Wu et al [14], which used maximal HR during an exercise test). Predicted HR was calculated by subtracting patient age from 220. CCI was then calculated for each participant as the ratio between their respective subtraction of peak, baseline HR values, and subtraction of predicted, baseline HR values.

### Parameters of the Simulation

**Figure 1:**
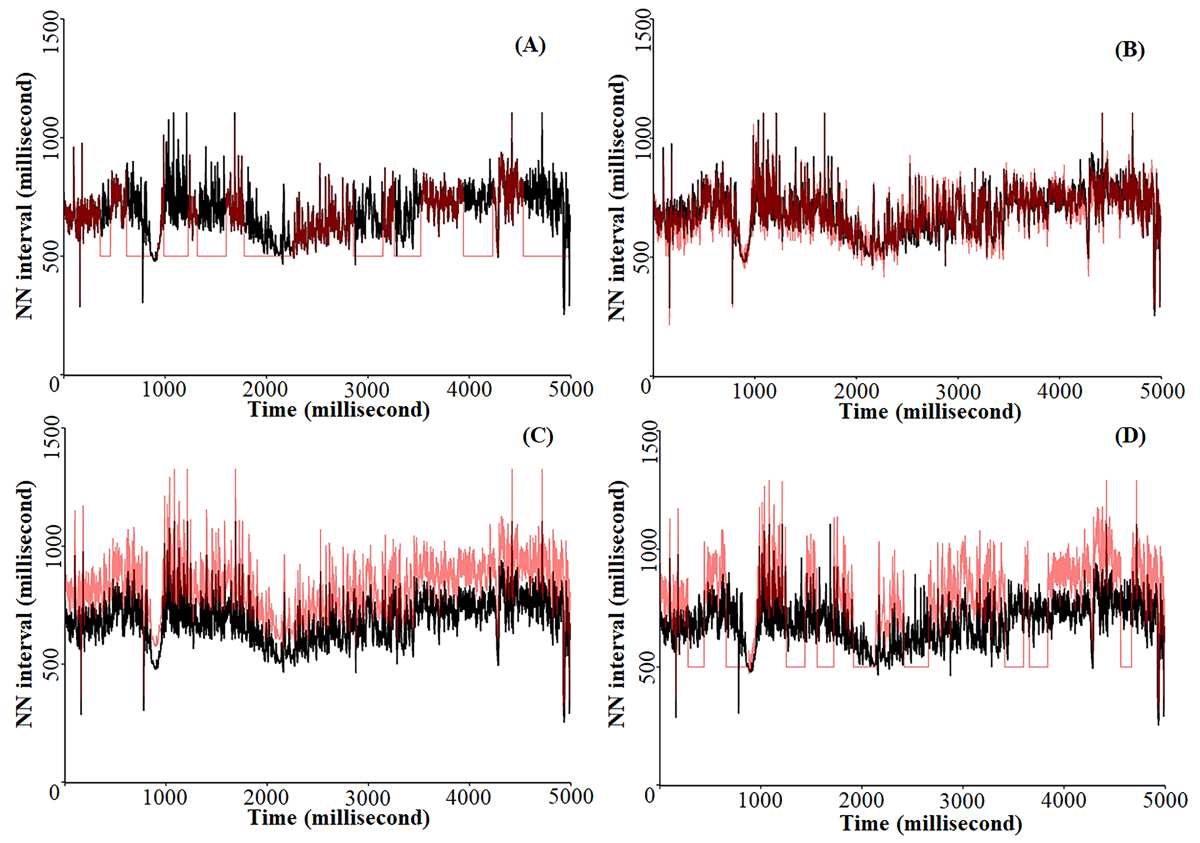
Visual depiction of 4 simulated perturbations for a typical participant, where the black line represents the unperturbed data and the transparent red lines represent the following perturbations: (A) 20% of data missing, (B) noise with magnitude equal to 20% of mean, (C) a positive bias of 20%, and (D) all 3 perturbations combined.

To simulate real-world conditions that may affect HR and activity monitor data, 3 perturbations were individually added to each participant’s raw data, including NNI, HR, and PMSC time-series data. HR data were extracted from raw NNI time-series data in the initial analysis steps. Postextraction, HR, and NNI data were analyzed separately adding the perturbations. First, gaps of varying length and frequency were added to the HR monitor and activity monitor data (Figure 1A). This perturbation was achieved by randomly selecting segments of HR, NNI, and PMSC values for periods of 1 to 5 minutes in length and removing them. The proportion of the entire data set that was removed was varied from 5% to 95% in increments of 1.2% (75 steps). In this analysis, HR and NNI values in the removed proportion of the data set were set to 35 beats per minute and 500 milliseconds, respectively. Secondly, flicker (ie, pink) noise in the HR monitor and activity monitor data was simulated using the power law noise generator function in R (R Foundation for Statistical Computing; Figure 1B). Next, the amount of noise added to HR, NNI, and PMSC values was varied between 0% and 150% (in 2% increments) of the mean value for that feature. After the noise was added to the time series, negative values for HR and NNI were transformed to positive by taking their absolute and then both HR and NNI values less than 1 were set equal to 1. For the third perturbation, a positive systematic bias ranging between 0% and 150% (in 2% increments) was added to the HR, NNI, and PMSC data (Figure 1C). PMSC values less than 0 were set equal to 0 after the addition of any perturbation. Finally, all 3 types of perturbations were combined in a final simulation, with each perturbation being applied at an equivalent level varying between 0% and 150% in increments of 2% for noise and bias, and between 5% and 95% in increments of 1.2% for missingness (ie, 75 steps for each of the 3 perturbations; Figure 1D). A total of 75 perturbed data sets were created for each participant and analyzed in this study.

### Prediction Models

Features were extracted for the unperturbed raw data and each of the data sets with a simulated perturbation. A total of 285 features (282 continuous and 3 categorical) were extracted from the HR, NNI, and PMSC time-series data for each participant. After variable preselection, 18 out of 285 features were reserved to predict the CCI. The remaining 267 features were eliminated from the analysis based on (1) low correlation with the CCI variable and (2) features that remained consistent across all data sets (perturbed and unperturbed) and as such were deemed uninformative. Pearson correlation coefficient was computed to quantify the association between the CCI and each feature and considered only features with *P*≤.10 for the analysis to filter out features weakly correlated with the CCI outcome. Next, the *rfcv* [B1] function from the *randomForest* library in R was used to create a random forest model predicting CCI in each data set, and to output 3-fold cross-validated prediction performances in the form of the root mean squared error (RMSE) between the actual and predicted CCI values. The default parameters were left unchanged for each of the developed random forest models, allowing for direct comparability. Given the small amount of data and the resultant variability in the results of a single 3-fold cross-validation, the assessment of prediction performance was repeated 25 times for each data set. The mean and standard error of the RMSE achieved by each model were then outputted and compared between each of the simulated conditions. Baseline RMSE (RMSE for a random classifier) was calculated by considering the mean of the actual CCI values as predicted and obtaining the standard deviation of the prediction errors. All analyses were performed using R (v4.1.1).

### Ethics Approval

Research ethics approval was not needed for this study as it is a secondary use of publicly available data. The original data for this study were collected with participant consent after approval from the Ethical Committee of the University of Pisa (#0077455/2018) [11,12].

## Results

**Figure 2:**
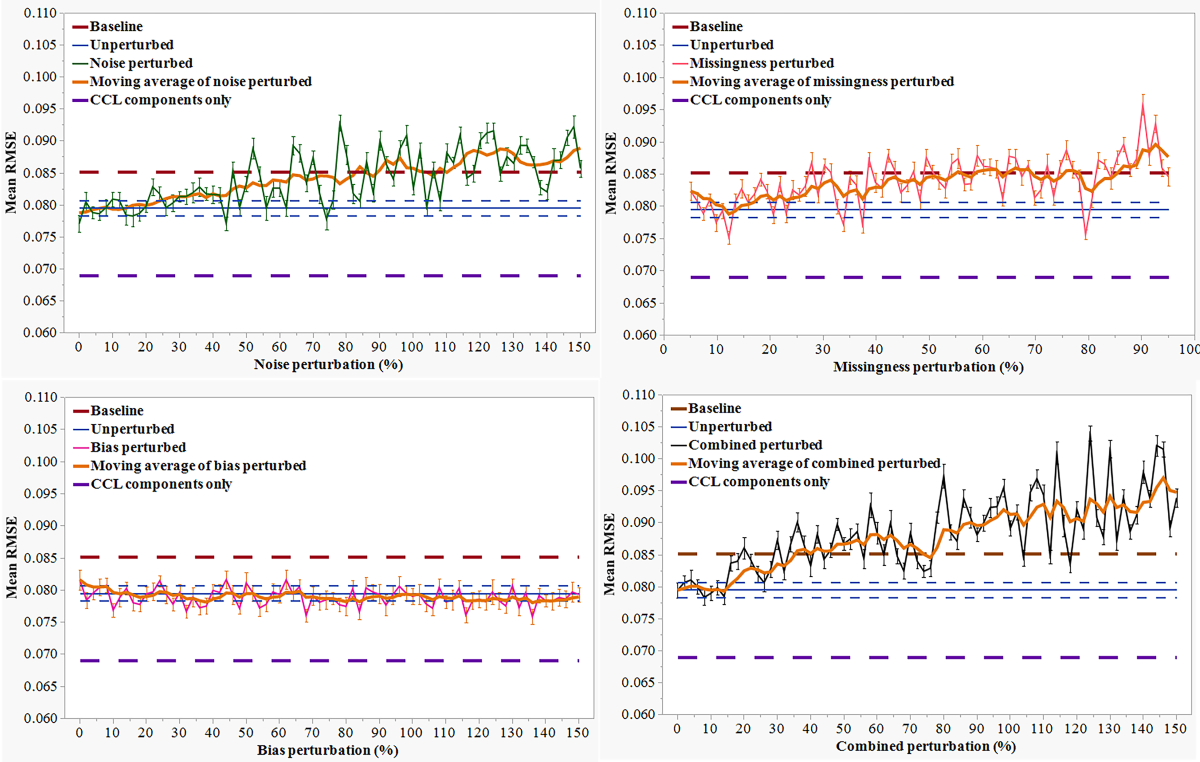
Progression of root mean squared error (RMSE) for prediction of cardiac competence index (CCI) using 3-fold cross-validated predictive performance over multiple iterations with increasing level of perturbations in source data (top left: noise; top right: missingness; bottom left: bias; and bottom right: combined). CCL: cardiac competence limit.

Prediction performance was assessed for a total of 76 conditions (1 unperturbed and 75 perturbed simulations). The prediction model for CCI, created using the unperturbed data set, achieved a mean RMSE of 0.079 (SD 0.001) versus a baseline RMSE of 0.085 (SD 0.001). RMSE for prediction model for CCI, when using the maximum and minimum HR terms included in the calculation of CCI, was 0.069 (SD 0.006). Both the noise perturbation and degree of missingness showed stability at the low end of the perturbation spectrum with increasing RMSE starting with medium-high degrees of perturbation. For the noise perturbation (Figure 2), the RMSE remained stable up to a 20% perturbation. Thereafter, RMSE increased up to an 80% noise perturbation at which point the RMSE was no better than the baseline model. A similar pattern was seen for missingness with a stable RMSE up to a 20% data missingness, degrading performance up to a 50% missingness and loss of predictive ability thereafter. Introducing bias in the time series had no effect on the RMSE at any level. The progression of RMSE for the combined perturbations showed a similar pattern as noise and missingness, with stability up to a 20% perturbation, followed by performance degradation and a loss of predictive ability at a ~35% perturbation.

## Discussion

### Principal Findings

In this study, we used continuous step count and HR data acquired using a standardized protocol and thoroughly validated wearable devices [15,16] and simulated the effect of data degradation on a predictive model. We showed that the performance of the prediction models for CCI remained stable up to a 20% feature degradation (for all types). Thereafter, model performance decreased with increasing perturbation of up to 40%-50% feature degradation, at which point the models were no longer predictive. Should these findings be replicated in other contexts, it follows that the moderate decrease in data reliability associated with consumer-grade wearable devices might not be a contraindication to the use of the data generated from these devices being used in prediction models.

### Methodological Considerations

The results from this study show that prediction models created using machine learning based on the features derived from time-series data can be relatively resistant to the low-moderate degree of degradation in the quality of the underlying data. There are numerous aspects of our simulation that warrant further discussion. First, we focused only on step count and HR as those are 2 of the most common types of data acquired through consumer-based wearables devices. Recently released devices now continuously measure other physiological indices such as pulse oximetry, breathing rate, or HR variability. Thus, the same type of simulation study will be needed for each of these measurements to establish the degree of reliability needed to maintain the performance of prediction models derived from these data. Second, we decided to run the simulations far beyond the range of perturbations that have been previously reported for these devices. We used this approach to both observe the behavior of the prediction models over the expected range of perturbations and the degree of perturbations needed for model’s performance to degrade up to the point where it is no longer predictive. Future studies assessing other physiological markers might not need to investigate behavior over such a large range given that the upper limit is clearly outside of the realistic range. Finally, it is important to note that the effect of feature degradation might not be the same with all machine learning methods that can be used to create prediction models. The ability of random forest models to effectively integrate continuous variables with highly abnormal distributions might make such models uniquely suited to resist the effects of degradation in the underlying features in a way that other methods could not replicate. Future studies will be needed to compare the effect of data degradation on different prediction models.

The use of machine learning to handle continuous monitoring data, particularly in contexts where data reliability and acquisition could be an issue, is appealing. In a traditional, probabilistic-based, predictive modeling approach, the accuracy and reliability of the predictors is highly important. However, with machine learning, the paradigm can be different. Machine learning uses secondary, data-derived features for predictions, and model performance is predicated on the stability and characteristics of those features and less so on the reliability of the underlying data. This is particularly true for time-series data, where features are created from multiple data points, something which tends to reduce their variability. Prediction models created using machine learning also have 3 additional major advantages in this context. First, many machine learning algorithms create higher-order features (features created through the combination of other features in a process akin to statistical interactions); meaning that the features predicting the outcomes are even further removed from the raw data, and thus, are less sensitive to small perturbations. Second, the ability of machine learning models to use higher-order features also allows for predictive models to use features specific to different segments of the population. Third, machine learning models are able to take into consideration far more features in making a prediction than their probabilistic counterparts. Thereby, data perturbations need to affect more features and be more pronounced to derail model performance, since the algorithm can still make predictions based on the more stable features.

### Comparison With Prior Work

There are 2 main challenges with the use of consumer-oriented devices to acquire data to be used for clinical applications—the accuracy of the data generated and the use of surrogate measurements. Previous studies of activity trackers have generally found limited accuracy in step count, the measurement of which is prone to substantial noise, particularly in high physical activity situations [5-8]. A systematic review on the subject has found that consumer-grade devices meet acceptable accuracy standards for step count half the time, overestimating higher intensity activity while underestimating medium intensity activity. Additionally, wrist-worn devices are more prone to high levels of noise and a greater amount of data missingness, given that the data acquisition is free-living and not standardized [17]. On the other hand, the accuracy of HR measurements, something much less sensitive to data perturbations, was found to be reasonably accurate albeit with an overall negative bias and lower accuracy when patients were not in sinus rhythm. For this metric, accuracy was lowest during high-intensity activity [18,19].

An additional common criticism of the use of commercially available wrist-worn devices for the clinical monitoring of patients is that the majority of devices do not directly measure the physiological features of interest but instead use surrogate, more easily measurable features to approximate or predict those features. For example, many wrist-worn devices do not measure HR directly. They use a method called photoplethysmography, which looks at rapid changes in red and green light absorption in the wrist to estimate HR [20]. Cardiac pulsations and the associated forward blood flow reflect red light and absorb green light, and consequently, there is less green light absorption in between heart beats. The speed of variation between red and green light absorption is used to estimate HR. The use of surrogate markers can have the following 2 drawbacks: first, the approximation or prediction can be associated with a higher error rate than a direct measurement and thus have lower reliability, and second, some important features might be missed. For example, in the case of HR measurements by photoplethysmography, since the device does not measure a full electrocardiogram signal, not all possible cardiac intervals can be assessed, which may result in missing clinically important features [17,21-23]. Moreover, HR detection with photoplethysmography is dependent on having a sufficient volume of ejected blood with each cardiac cycle (ie, perfusion) to generate a detectable flow in the extremity where the device is worn. In some heart rhythms and in some heart conditions, stroke volume may be inadequately low for detection in certain cardiac cycles leading to the underestimation of HR [24,25].

The use of data acquired from wearable devices in prediction models is still relatively new, so much so that a systematic review found only 8 published models based on wearable data between 1997 and mid-2019 [26]. In a recent systematic review of wearable sensors used in Parkinson disease in a clinical area with a long history of sensor-based monitoring, only 7 out of 74 studies reported the creation of prediction models from these data. The development of those models was dependent on the use of increasingly complex machine learning algorithms that required the combination of diverse sets of higher-order features to achieve a high performance [10]. The same trend toward increasing the use of machine learning has been observed in the area of continuous glucose monitoring for patients with diabetes [27,28]. None of those previous investigations addressed the potential effect of data accuracy and data degradation on the performance of prediction models.

### Study Limitations

This study must be considered in light of some limitations. First, given that the outcome (ie, CCI) and predictive features were derived from the same HR and physical activity data, it is possible that the prediction models may overperform compared to prediction models for outcomes not derived from the underlying data. Additionally, it is worth noting that the calculation to obtain the CCI was modified based on the data available in this study, and, as such, it is only an approximation of true CCI. Second, the limited sample size from a single data set and the single modeling approach used here preclude treating this study as definitive. That being said, this study provides a proof of concept aimed at raising an important question related to the reliability of the data obtained from consumer-grade monitoring devices. Future studies will need to focus on large cohorts, on more remote outcomes, and on exploring different modeling strategies that may be more or less resistant to the degradation of source data.

### Conclusions

In conclusion, in this proof-of-concept study, we showed that the performance of predictive models for CCI generated from continuous devices is relatively stable to degradation in the quality of the underlying data. This finding, while needing confirmation in larger studies, suggests that by itself, lower accuracy of measurements may not be an absolute contraindication for the use of data from consumer-oriented monitoring devices in prediction models intended to inform clinical management.

## Abbreviations

CCI: cardiac competence index
HR: heart rate
MMASH: Multilevel Monitoring of Activity and Sleep in Healthy People
NNI: N-N interval
PMSC: per-minute step count
RMSE: root mean square error

## Data Availability

All data used for this study are publicly available from the PhysioNet database [11,12].
